# Co-infection alters population dynamics of infectious disease

**DOI:** 10.1038/ncomms6975

**Published:** 2015-01-08

**Authors:** Hanna Susi, Benoit Barrès, Pedro F. Vale, Anna-Liisa Laine

**Affiliations:** 1 Metapopulation Research Group, Department of Biosciences, University of Helsinki, PO Box 65 (Viikinkaari 1), Helsinki FI-00014, Finland; 2 Centre for Immunity, Infection and Evolution, School of Biological Sciences, University of Edinburgh, Edinburgh EH9 3FL, UK; 3 Institute of Evolutionary Biology, School of Biological Sciences, University of Edinburgh, Edinburgh EH9 3FL, UK

## Abstract

Co-infections by multiple pathogen strains are common in the wild. Theory predicts co-infections to have major consequences for both within- and between-host disease dynamics, but data are currently scarce. Here, using common garden populations of *Plantago lanceolata* infected by two strains of the pathogen *Podosphaera plantaginis*, either singly or under co-infection, we find the highest disease prevalence in co-infected treatments both at the host genotype and population levels. A spore-trapping experiment demonstrates that co-infected hosts shed more transmission propagules than singly infected hosts, thereby explaining the observed change in epidemiological dynamics. Our experimental findings are confirmed in natural pathogen populations—more devastating epidemics were measured in populations with higher levels of co-infection. Jointly, our results confirm the predictions made by theoretical and experimental studies for the potential of co-infection to alter disease dynamics across a large host–pathogen metapopulation.

## Abstract (web summary)

Co-infection of plants with multiple pathogen strains is predicted to alter disease dynamics. Here, Susi *et al.* use experimental and natural population data to show that co-infected host plants spread more disease and cause more devastating epidemics than singly infected hosts.

## Graphical abstract

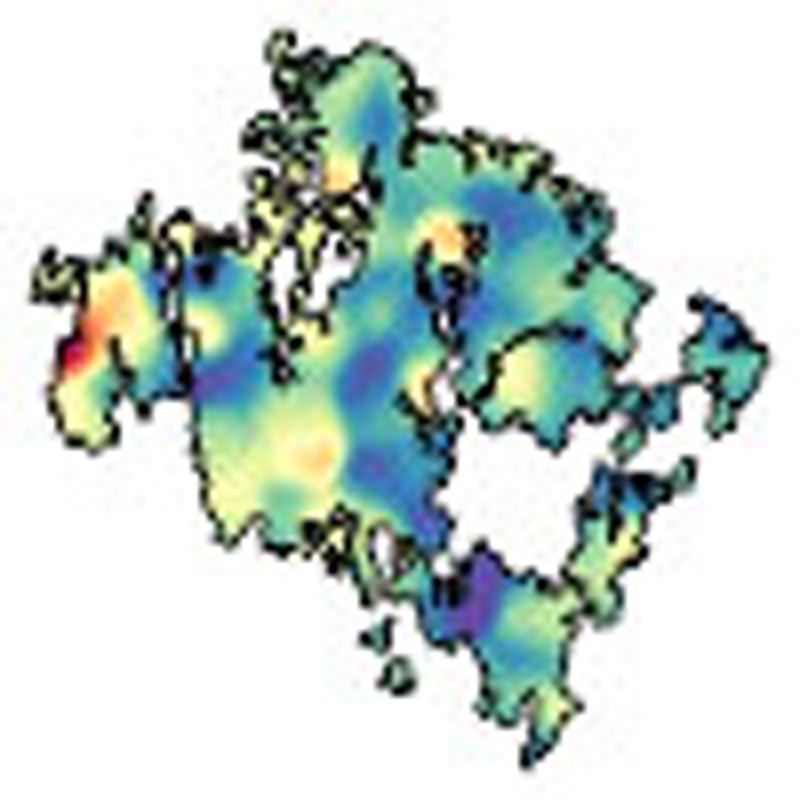

A long-standing challenge in studies of pathogen evolution and epidemiology has been to link within- and between-host levels of disease dynamics^1^. William Hamilton^2^ first proposed that diverse parasitic infections should favour more virulent genotypes, as competition for limited host resources results in a ‘tragedy of the commons’ situation where non-optimal levels of host exploitation may emerge (that is, the trade-off hypothesis^3^). Hence, within-host disease dynamics are proposed to change under co-infection, and this assumption is at the heart of many theoretical models^3^,^4^ predicting increased virulence when multiple strains simultaneously infect the same host. Given the often inconspicuous nature of the parasitic lifestyle and lack of morphological differentiation among strains, the empirical studies of disease dynamics under co-infection have lagged behind the theoretical predictions. However, with molecular tools becoming increasingly available for the study of parasites^5^, we now know that co-infections are common in the wild^6^,^7^,^8^, and may change the within-host disease dynamics^9^,^10^.

Changes in within-host infection dynamics under co-infection^11^,^12^ may have profound consequences for between-host dynamics and epidemiological outcomes. Empirical studies suggest that within-host competition and pathogen transmission are tightly linked, resulting in the potential for changed rates of disease transmission under co-infection^9^,^13^. Moreover, the arrival sequence of co-infecting pathogens may have an effect on co-infection dynamics. In some studies, the first arriving genotype has an advantage over later arriving ones^11^,^14^. This may be due to the benefit of earlier colonization, or the outcome may be mediated by systemic-induced resistance of the host where the first arriving pathogen induces an immune response. Hence, the outcome of within-host co-infection may be mediated by the host’s responses to infection^15^,^16^,^17^. Together these results suggest that co-infection is a potentially powerful driver of pathogen evolution and epidemiology. The applied implications of co-infection range from resistance priming^18^ whereby host sensitivity to infection is manipulated by priming its immune system, to other epidemiological interventions and virulence management^19^. A precise understanding of how within-host interactions translate to disease dynamics at the population level is therefore urgently needed^12^. Moreover, to date, remarkably little is known about how levels of co-infection vary spatially across host populations^20^.

We focused our study on the obligate fungal pathogen *Podosphaera plantaginis* naturally infecting the host plant *Plantago lanceolata*. The visually conspicuous symptoms caused by *P. plantaginis* enable accurate tracking of infection both in the wild and under experimental conditions. Twelve years of epidemiological data across a large host population network in the Åland Islands, southwest of Finland, have demonstrated this pathogen to persist as a highly dynamic metapopulation^21^. Co-infections, whereby two or more strains of *P. plantaginis* simultaneously infect the same host, are common in the Åland metapopulation^8^. In the wild, local host populations support considerable diversity in their resistance^22^, and hence, understanding to what degree the dynamics of co-infection are mediated by the host is critical for understanding how general the changes in epidemiological dynamics in response to co-infection may be in the natural populations.

Our experimental results show that within- and between-host disease transmission rates are altered by a change from single strain infection to co-infection. Moreover, the effects of co-infection we observe under experimental conditions can also impact epidemiological dynamics across a large natural host–pathogen metapopulation. Hence, accounting for co-infection may be central for successful disease prevention efforts.

## Results

### Phenotyping host and pathogen lines

Before the common garden experiment, we characterized the resistance and infectivity phenotypes of 41 host and seven pathogen lines, respectively, using an inoculation approach^23^ (Methods). Qualitatively resistant plants blocked infection by most of the tested pathogen strains (Supplementary Table 1) but were susceptible to at least one of the two pathogen strains used in the experiment (Supplementary Table 1). Quantitative resistance was estimated from pathogen aggressiveness measures, selecting those hosts that supported slow pathogen development rates and low sporulation. Average pathogen sporulation was ≤2.5 (Bevan’s score, see Methods) in the hosts classified as quantitatively resistant (Supplementary Table 1). Two pathogen strains—3 and 10—were selected for the experiment on the basis of their infectivity profiles that matched the range of plant genotypes in the experiment (Supplementary Table 2). We chose allopatric host–pathogen combinations originating from different populations for the common garden plots so as not to confound our results with potential local adaptation^24^.

### Common garden experiment

**Figure 1:**
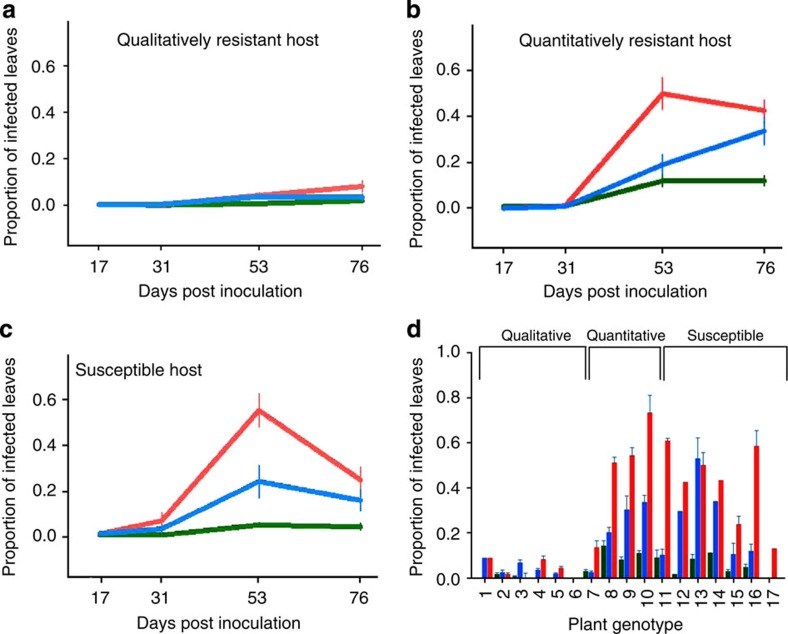
Co-infection increases disease prevalence. *P. plantaginis* epidemics in common garden *P. lanceolata* plots representing qualitative (**a**), quantitative (**b**), and susceptible (**c**) resistance strategies. Disease severity, measured as proportion of infected leaves, is shown at 17, 31, 53 and 76 days post inoculation (DPI) for the three pathogen treatments: strain 3 singly (green), strain 10 singly (blue) and co-infection of strains 3 and 10 (red). (**d**) Mean disease severity across *P. lanceolata* genotypes measured at the highest peak of epidemics (53 days after inoculation) comparing single infections (3=green and 10=blue) to co-infection treatment (red). Error bars are based on s.e.m.

To determine the effect of co-infection on within- and between-host transmission, we set up common garden plots of *P. lanceolata* representing the different host resistance strategies—qualitative and quantitative resistance, and susceptibility—characteristic of the interaction between these species. In brief, qualitative resistance operates by preventing infection establishment, whereas quantitative resistance mitigates the development of established lesions (for details, please see Methods). We found that co-infection amplified disease spread in the common garden plots, even though the co-infection treatment received the same total dose as the singly infected plots (Fig. 1a–c and Table 1). Disease prevalence (measured as the proportion of infected leaves per plant) was consistently higher in the co-infected than in the singly infected treatments across all the three host resistance types (Fig. 1a–c and Table 1). Furthermore, across all plant genotypes categorized as susceptible and quantitatively resistant, disease load was higher at the peak of epidemics (53 days post inoculation (DPI)) in the co-infected plots compared with the singly infected plots (Fig. 1d and Table 2). No clear differences were observed in the qualitatively resistant plant genotypes where disease levels were overall low (Fig. 1d). In the singly infected plots, strain 10 always achieved higher prevalence compared with strain 3 at the peak of the epidemic (Fig. 1a–c and Table 1). Genotype analysis of infections from the co-infected plots revealed that the prevalence of strains 3 and 10 differed between susceptible and quantitatively resistant plots at the end of epidemics (G-test: *G*=52.48; *P*<0.0001), with strain 3 outperforming strain 10 in quantitatively resistant plots, while strain 10 was more successful in susceptible host plots.

### Spore-trapping experiment

**Figure 2:**
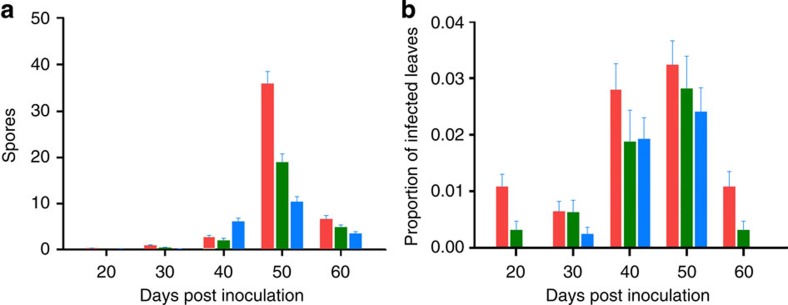
Co-infection increases spore release and pathogen transmission. Singly inoculated plants shown in blue and green and co-inoculated plants in red. (**a**) Mean number of spores caught on microscope slides from singly inoculated (3=green and 10=blue) and co-inoculated (red) plants. (**b**) The proportion of live leaf traps that became infected. Error bars are based on s.e.m.

The increase in disease prevalence that we observed at both host genotype and plot levels under co-infection suggests higher transmission rates under co-infection^25^. To test this hypothesis, we set up a spore-trapping experiment, where we inoculated individual plants using the same pathogen treatments (always the same total dose consisting of strain 3, strain 10 or both) and monitored transmission throughout the growing season by trapping spores on both microscope slides (to quantify pathogen spore shedding), and live leaf traps that allow for disease establishment (to quantify successful establishment). Co-infected plants released more spores than singly infected plants (generalized linear mixed model: Pathogen treatment × time; F_2,334_=25.88; *P*<0.0001; Fig. 2a and Table 3), with the difference being most pronounced at the peak of the epidemic season on 50 DPI (generalized linear mixed model: F_1,66_=18.80; *P*<0.0001). Infection establishment on the live leaf traps was also higher from co-infected hosts than singly infected hosts (generalized linear mixed model: F_1,338_=4.81; *P*=0.0290; Fig. 2b and Table 4).

### Disease dynamics in the wild

**Figure 3:**
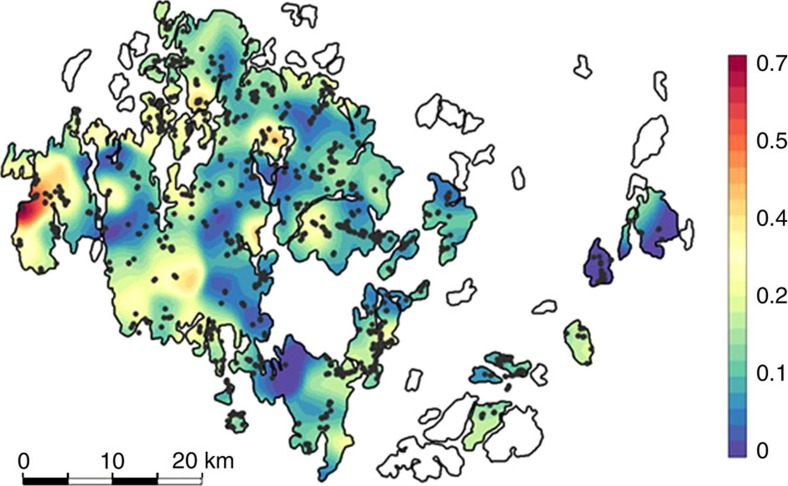
Co-infection is common in natural populations. Map of the relative probability to detect co-infection versus single strain infection in the *P. plantaginis* metapopulation in the Åland Islands in 2012. The smoothing bandwidth of the kernel was set to 1,258 m (the estimated range obtained with the spatial Bayesian logistic regression model). The probability of detecting a co-infected host is indicated by the colour scale, from blue (low probability of co-infection) to red (high probability of co-infection). Sampling sites are shown by grey points.

Our experimental infections suggest that co-infection is an important driver of disease dynamics in the wild. To test this, we first assessed the prevalence of co-infection in the large natural metapopulation of *P. plantaginis,* and then measured how the level of co-infection was associated with the severity of epidemics. We sampled 641 *P. plantaginis* populations from its natural distribution in the Åland archipelago, southwest of Finland in September 2012 (Fig. 3). Our SNP genotyping protocol allowed us to estimate the number of multilocus genotypes (MLGs) and prevalence of co-infection within pathogen populations^8^. Co-infection of host plants proved to be common across the *P. plantaginis* metapopulation (Fig. 3; Supplementary Fig. 1), with 53% of the pathogen populations having at least one infection consisting of two or more strains of *P. plantaginis*. In these populations, prevalence of coinfection was highly variable, ranging between 10 and 100%. We found that prevalence of co-infection was higher in well-connected pathogen populations suggesting that spatial structure of the pathosystem can have a strong impact on disease dynamics (Table 5).

**Figure 4:**
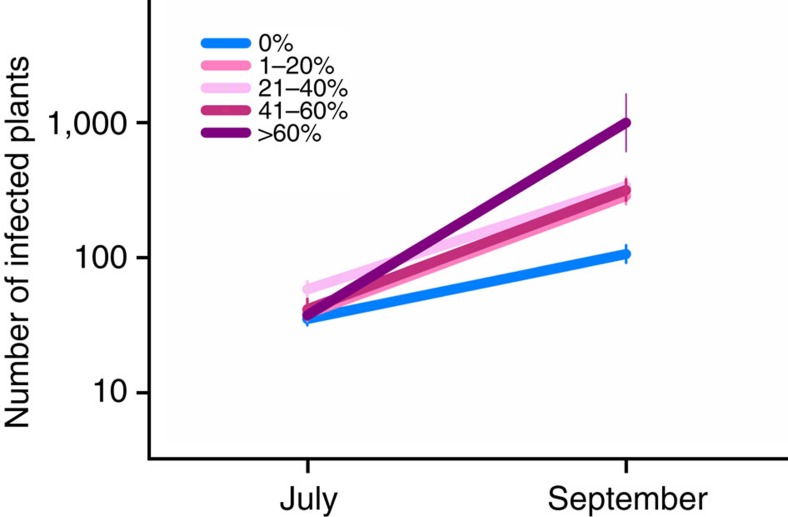
Co-infection causes a rapid increase in epidemics. The increase in pathogen population size between July and September measured as the number of infected host plants in populations with varying levels of co-infection. The co-infection level of the 135 populations is shown by different colours as indicated in the legend. Error bars are based on s.e.m.

To test whether the prevalence of co-infection (as measured at the epidemic peak in September) affects disease spread within host populations, we measured pathogen population growth rate from July to September in 2012 in 135 *P. plantaginis* populations (Methods). We found that more devastating epidemics were observed in populations where co-infection was more prevalent (generalized linear mixed model: *χ*^*2*^=6.88; *P*=0.0087; Fig. 4). This was the case even after controlling for MLG diversity and connectivity among pathogen populations, which may strongly correlate with spore immigration into local populations^26^.

## Discussion

Our study provides conclusive experimental evidence that within-host disease dynamics change under co-infection resulting in an increase in between-host disease transmission. Specifically, these results provide a clearer understanding of how co-infection changes disease load across host genotypes (in experimental plots; Fig. 1d), and how spore shedding changes through time under co-infection (in the spore-trapping experiment; Fig. 2). These results confirm that the response to co-infection in *P. plantaginis* can have an immediate impact on the epidemiological dynamics^10^ leading to increased release of spores and subsequent transmission. Critically, the effect is so powerful that within a natural host–pathogen metapopulation it was possible to observe more devastating epidemics in local populations where co-infection was more prevalent (Figs 3, 4).

The mechanism underlying the observed effect of co-infection is unclear, but given that the inoculum dose is identical across the treatments, differences in spore shedding are likely due to interactions between strains. For an epiphytic parasite such as *P. plantaginis*, a previously infected area of host tissue is not readily available for colonization for other spores. Hence, the spatial distribution of pathogen strains across the host surface is of great importance and can generate considerable competition between strains. Evidence for these interactions between strains is apparent in the common garden experiment, as there was no consistent benefit to either strain in the co-infection treatments but instead strain dynamics changed according to host resistance and genotype. The host genotype-specific outcome of co-infection is important to consider in the context of genetically variable metapopulations in the wild, where local populations support considerable diversity in resistance^22^. Further, we find that not only is co-infection common in natural plant populations, but severe epidemics also coincide with high prevalence of co-infection (Figs 3, 4). These results from a natural metapopulation suggest that the changes that were observed in the common garden population are also important in the wild.

Our experimental design included plants of different genotypes to understand whether the outcome of co-infection varies among different host resistance strategies. As in most plant–pathogen interactions^27^, the first step of resistance in *P. lanceolata* is strain specific, with the same host genotype expressing resistance against some strains (that is, recognition) of the pathogen while being susceptible to others (that is, non-recognition)^22^. Even on susceptible hosts, the development of an established strain may still be mitigated by quantitative host resistance^23^. A priori, we expected to see the smallest changes in disease dynamics in response to co-infection on the qualitatively resistant hosts that are expected to effectively block infection altogether, and, as expected, disease levels were overall low on the qualitatively resistant hosts (Fig. 1). On the quantitatively resistant hosts, the first infection may induce host responses that mitigate the development of subsequent strains^14^, leading to lower disease levels than those measured on the susceptible hosts under co-infection. However, quantitatively resistant genotypes (as well as susceptible genotypes) showed high levels of disease load (Fig. 1) and higher pathogen spore shedding at the peak of epidemics (Fig. 2), suggesting that quantitative resistance becomes ineffective over the course of the epidemic.

The results we present here are in line with work in mammalian hosts. For example, Lass *et al.*^28^ found that mice co-infected with a bacterial pathogen and a gastrointestinal helminth shed markedly higher numbers of helminth eggs, and had higher bacterial loads compared with single infections. In fact, some individual mice in that study were classified as super shedders due to shedding significantly higher numbers of helminth eggs than average over the course of the experiment. That work, therefore, supports our view that co-infection can be an important driver of epidemiological dynamics. Thus, our results could have implications for predictive epidemiology^25^ by unveiling which diseased individual hosts should be targeted in disease control programs. If resources are limited and only some individuals can be treated or removed, it would be most advantageous to target hosts that are co-infected. This points towards a need for a quick and easy method of genotyping infection loads from individual hosts.

Certainly co-infection is only one factor affecting individual host variation in disease transmission, and a myriad of other factors are likely to generate variation in within-population transmission rates in nature^29^, including climatic differences^30^,^31^, host genotypic effects^32^, host nutrition level^33^, host sex^34^,^35^ and even the type of anti-pathogen therapy^36^,^37^ used. Establishing direct links between the fundamental axes of host resistance variation and realized epidemiological dynamics in single infection versus co-infection scenarios offer an exciting future avenue of research, and is needed to truly consider the role of co-infections when designing epidemiological interventions or virulence management efforts^29^,^38^.

## Methods

### Field surveys and sampling of infection

*Plantago lanceolata* is an outcrossing perennial herb that is naturally infected by *P. plantaginis*, a powdery mildew fungus (Ascomycota). This pathogen is a host-specific obligate biotroph that completes its entire life cycle on the surface of the host plant where it is visible as localized (nonsystemic) white powdery lesions. The pathogen is a significant stress factor for its host and may cause host mortality^22^. Approximately 4,000 *P. lanceolata* populations have been systematically mapped in the Åland Islands, southwest of Finland, since the 1990s. The epidemiological dynamics in these populations have been studied since 2001 (refs 26, 39), demonstrating that *P. plantaginis* persists as a highly dynamic metapopulation through extinctions and (re-) colonizations of local host populations^21^. The first visible signs of infection appear in late June as white-greyish lesions consisting of mycelium supporting spores (conidia) are formed. Some six to eight clonally produced generations (estimated from spore germination-production times observed in the laboratory) follow one another in quick succession, often leading to a local epidemic with a substantial proportion of the host individuals being infected by late summer^40^. Resting spores (chasmothecia) appear towards the end of the growing season from August to September.

To measure how disease prevalence changes within host populations during epidemics, we visited all the 283 pathogen populations that had been identified in September 2011, in July 2012 and again in September 2012. In total, 144 populations were infected at both the visits. In both the surveys, we scored pathogen population size on a categorical scale (0=absence of mildew, 1=1–9 infected plants, 2=10–99, 3=100–999 and 4=1,000 or more). These data were used for the analysis of change in disease prevalence as a function of co-infection (see Statistical Methods for details). In addition, in September 2012, one infected leaf per infected plant was sampled for genotyping (*N*=641). Up to 10 samples per population were collected, with a minimum distance of 5 m between infected plants. The infected leaves were placed in separate falcon tubes and brought back to the laboratory where fungal material for each sample was collected by scraping off the surface of the infected leaf. This material and a 1 cm^2^ piece of the same infected leaf were placed in an individual well of a 96-well plate. Samples were stored at −20 °C until DNA extraction (see below).

### Inoculation experiments

We set up an inoculation experiment to measure the resistance and pathogenicity phenotypes of hosts and pathogens to select host and pathogen genotypes for the common garden populations described below. Altogether 28 strains of *P. plantaginis* were collected as infected leaves from six natural pathogen populations in the Åland Islands (population IDs 877 (two strains), 689 (four strains), 228 (two strains), 2,821 (three strains), 9,066 (two strains) and 9,609 (eight strains)) in 2010, and placed on Petri dishes on moist filter paper. Genetically homogenous strains were obtained by repeating at least three single-colony inoculations^41^. The strains were maintained in Petri dishes on fresh leaves of *P. lanceolata* in a growth chamber at 20±2 °C and a 16 light/8 dark photoperiod, and transferred to fresh leaves every 2 weeks. As some strains were lost during purification and maintenance, seven strains were used for the experiment (Supplementary Table 2). Repeated cycles of inoculations were performed before the experiments to obtain adequate stocks of sporulating fungal material.

Host plants were collected as seeds from seven natural *P. lanceolata* populations in Åland (IDs 4 (eight plants), 325 (one plant), 511 (seven plants), 1,062 (four plants), 1,413 (eight plants), 2,220 (eight plants) and 9,031 (five plants)) and stored at room temperature. Seeds were germinated by placing them in 0.8 l pots in a mix of sand and potting soil (1:1) in greenhouse conditions of 16 h of light at +22 °C. Altogether 41 plants, originating from seven natural populations, were used in the inoculation experiment (Supplementary Table 1).

The inoculation study consisted of 287 host genotype–pathogen genotype combinations. Detached leaves of the host plants were placed on Petri dishes and challenged with conidia from a sporulating colony of ~1 cm in diameter by evenly brushing the spores over the exposed leaf. The development of lesions was followed daily from 4 DPI until 12 DPI using a dissecting microscope. Mycelium growth and sporulation rates were scored using Bevan’s scale^42^, ranging from 0 to 4 (0=no mycelium, 1=mycelium only, 1.5=mycelium producing very few conidia and colonies visible only under a dissecting microscope, 2.5=mycelium and sparse sporulation visible only under a dissecting microscope, 3=abundant sporulation and lesion size <0.5 cm^2^, 4=abundant sporulation and lesion size >0.5 cm^2^; ref. 42). Time to germination was defined as the first day that fungal mycelia were observed, and time to sporulation was defined as the first day that conidia were observed. The inoculation outcome was considered infective (1) for Bevan’s scores greater than 1, and non-infective (0) for category 0. All inoculations (21.4%) that did not produce any pathogen growth in 12 days were repeated. The resistance profiles of the plants and the infectivity and aggressiveness (germination and sporulation times, and Bevan’s score) profiles of all strains are presented in Supplementary Tables 1 and 2, respectively.

Six host genotypes were selected for the qualitatively resistant populations, four for the quantitatively resistant populations and seven for the susceptible populations. Two pathogen strains were chosen for the experiment; strain 3 from population 877 and strain 10 from population 2,821, as they were infective on the range of host genotypes required for the experiment.

### Common garden experiment

We set up common garden plots at the Lammi Biological Station (61°05′28′′N, 25°03′90′′E) to measure how co-infection and host resistance strategy jointly shape disease transmission under semi-natural conditions. Neither *P. lanceolata* nor *P. plantaginis* occur naturally in this area, and hence inoculum coming from outside was highly unlikely. The common garden experiment was established on a field where the topsoil layer was removed and replaced with a mixture of garden soil and sand. Before the experiment, each plant genotype was cloned in the greenhouse according to methods described in^22^ yielding up to six clones representing each host genotype (Supplementary Table 1). The 8-week-old ramets were placed outside for 1 week of acclimation until the common garden plots were established in May 2011.

We planted 24 0.5 m^2^ plots each consisting of 10 plants. Each plot consisted of plants belonging to the same resistance category. Each resistance category and pathogen treatment combination, including the controls, was replicated twice. Plants belonging to the same resistance category were randomly assigned to one of the plots, and locations of the plants within the plots were also randomized. The plots were planted in the field in a 4 × 6 design with narrow paths separating the rows (Supplementary Fig. 2). We used three pathogen treatments in the plots: strain 3 alone, strain 10 alone and strains 3 and 10 together. The same pathogen treatment was applied to the same row to minimize contamination risk. Given the small distances separating the plots (Supplementary Fig. 2), no microclimatic differences are expected to take place at this scale. The inoculations were carried out by placing two diseased transmission plants within the common garden plots. The transmission plants were cloned from a genotype expressing broad susceptibility, which is utilized in laboratory maintenance of multiple strains of *P. plantaginis*. The source plants supported equal amount of inoculum of two 1 cm^2^ lesions so that the co-infected plots received half of the inoculum of each genotype, yet the same amount of inoculum altogether. We used 100-cm high plastic dividers between the plots to prevent disease spread among the treatments. The control plots without pathogen inoculum were monitored throughout the growing season to ensure that there was no disease spread between the plots. Genotyping of four to five samples (collected as described below) from each of the singly infected plots further confirmed that there was no spore movement among the plots: Only the pathogen strain used to inoculate each plots was recovered by genotyping at the end of the epidemic.

After the inoculations, the epidemics were allowed to develop naturally and disease dynamics were monitored by counting the number of diseased leaves in each plant 17, 31, 53 and 76 days after first inoculation. Day 17 post inoculation was chosen as the starting date as at this stage, the developing infection becomes visible to the naked eye. Subsequent surveys were carried out at 2–3-week intervals adjusting for weather conditions, as the infection is difficult to score reliably in the rain. By 76 DPI, the disease spread has seized as the temperature becomes unfavourable to the pathogen, and the host leaves begin to wither. The total number of leaves for each plant was counted in early July and again 76 days after first inoculation. The first count was used to calculate the infection prevalence for 17 and 31 DPI, and the last count for 53 and 76 DPI. At the end of the epidemic season in September, we collected three infected leaves from each plant from the co-infected plots for subsequent genotyping to determine whether the plants were infected by strain 3, 10 or both. In the laboratory, fungal tissue was scraped as a pooled sample of the three leaves representing each plant and placed into a micro tube together with a 1 cm^2^ piece of leaf and stored in −80 °C until DNA extraction.

### Spore-trapping experiment

To determine the release of powdery mildew spores and actual transmission rate in singly and co-infected plants, we performed a spore-trapping experiment under common garden conditions in the summer of 2013. In the experiment, we used four of the susceptible and four of the quantitatively resistant plant genotypes (genotypes 7–11, 13, 15 and 17 in Supplementary Table 1), with each genotype cloned into 14 replicates. The plants were placed in 11-by-11 cm pots at 1 m distances from each other in the field, and inoculated in mid-July with strain 3, strain 10 or both strains simultaneously. The amount of inoculum (all spores brushed off from a 1 cm^2^ 10-day-old sporulating lesion) was the same for all plants, with the co-infected plants receiving half of the dose of the single genotype inoculum (that is, all spores from a 0.5 cm^2^ lesion). Control plants with no inoculation confirmed that there was no spore movement between the plants. Four replicates of each plant genotype × pathogen treatment and two replicates of each plant genotype and control treatment were used resulting in 112 plants. The infection status of plants was monitored at 20 DPI and the 76 plants (79.2%) that had become infected were used in the analysis. Spore trapping was performed at 20, 30, 40, 50 and 60 DPI using two spore-trapping methods: four Vaseline-coated microscope slides attached to wooden sticks at 5 cm distance from the ground placed between the infected leaves and 16 detached *P. lanceolata* leaves of a known susceptible genotype attached to moist floral foam at 5 cm distance around the focal plant. The trapping period lasted 24 h after which the traps were removed. The Vaseline traps were then kept at 5 °C and subsequently examined under a microscope using four 25-mm transect lines to count the released spores. The live leaves were placed on moist filter paper in a Petri dish and kept in a growth chamber for 14 days after which their infection status (0/1) was checked.

### Genetic analyses

DNA extraction was performed using E.Z.N.A. Plant DNA kit (Omega Bio Tek Inc. Norcross, GA, USA) at The Institute of Biotechnology (Helsinki, Finland). Samples were genotyped with 27 SNP markers using the Sequenom MassARRAY iPLEX platform as described in ref. 8 at the Finnish Institute for Molecular Medicine (Helsinki, Finland). Automatic calling of the genotypes was performed using MassARRAY Typer 4 Software (Sequenom, San Diego, CA, USA). Because of the presence of null alleles in the studied populations, eight SNPs were discarded from the analysis. Out of the 5,402 genotyped samples, 5,153 (95.4%) have no missing data for the 19 remaining SNP markers. This data set was used to perform further analyses. The genotyping was used to identify the different MLGs of pure strains and to detect co-infection in the collected samples. *P. plantaginis* is haploid, and therefore the detection of a heterozygote genotype for one or more SNP markers is a clear indicator of co-infection^8^. We confirmed the reliability of the SNP genotype calling by duplicating 132 of the samples. Each of the 35 samples, where co-infection was detected, gave concordant results for the duplicates. Furthermore, the other 97 samples consisting of pure strains all resulted in the same MLG as previously.

The same DNA extraction and genotyping methods were used to distinguish between the two different strains and to identify co-infection at the plant level in the common garden populations. The pathogen strains used in common garden populations (strains 3 and 10) differ at eight loci used in the genotyping panel. A total of 54 pooled samples were genotyped for this experiment, each of them consisting of three leaves per infected host plant. The prevalence of the pathogen strains at the end of the epidemic was scored as MLG 3 and MLG 10 or ‘co-infection’ if one allele or both alleles were detected in a sample, respectively.

### Statistical analyses

We used the framework of generalized linear mixed models^43^, fitted with procedure GLIMMIX in SAS 9.2 (ref. 44) to analyze data from the common garden experiment and spore-trapping experiment. Data on proportion of infected leaves were arcsin-transformed before the analysis to achieve homoscedasticity and normality of residuals. For data on proportion of the trap leaves infected and the number of spores trapped in the spore-trapping experiment, we assumed a Poisson error distribution with a logit link function. First, we analysed the proportion of infected leaves during the growing season in the common garden populations with resistance type, plant genotype nested under resistance type, pathogen treatment and DPI as explanatory variables with a repeated measured model. We then analysed the proportion of infected leaves at the plant genotype level at 53 DPI (peak of epidemic) in single versus co-infected treatments by comparing co-infected plants against the single infection treatment, which resulted in higher disease burden across the plant genotypes. The purpose of this analysis was to test whether the higher disease load in co-infected plots was caused by higher disease load also at the host genotype level, or the summed performance of strains 3 and 10 across the different genotypes. Resistance type, plant genotype nested within resistance type and pathogen treatment were defined as model variables. In both the models, ‘plot’ was defined as a random effect.

We then analysed the effect of co-infection versus single infection on spore release and transmission in the spore-trapping experiment. The number of spores caught on each microscope slide and the proportion of infected trap leaves around focal plant were defined as response variables with pathogen treatment (co-infected or singly infected), plant genotype and DPI defined as explanatory variables.

In our analysis of co-infection in the natural pathogen metapopulation assessing the prevalence of co-infection with respect to sampling effort, we computed the relative risk surface of co-infection versus single infection across Åland using kernel smoothing. The estimation of the relative risk surface was performed by a Nadaraya–Watson type kernel smoother using the ‘spatstat’ R package function ‘relrisk’^45^. The smoothing bandwidth of the kernel was set to 1,258 m according to the estimated range obtained with the best spatial Bayesian logistic regression model.

To identify which factors affect prevalence of co-infection in pathogen populations, we modelled the number of co-infected samples detected in each population with a spatial Bayesian logistic regression model. The model included factors that have previously been identified as important for the dynamics of this pathogen^21^,^26^; distance to the shore, host population area, road presence, age of the population (binary variable: ‘newly infected population’/‘population already infected in 2011’), pathogen-based connectivity (as described in ref. 46), the number of MLGs identified in each population and disease abundance in the population in September 2012. The number of co-infected samples relative to the total number of samples in a population was used as the response variable with a binomial error distribution. The spatial covariate was considered as a random effect to account for any potential bias of spatial autocorrelation while the other factors were included as fixed effects. Several models were considered. The first model was fitted only with the fixed effects, which is equivalent to an ordinary logistic regression model. We also fitted a model only taking into account the random spatial effect. Each investigated factor was first fitted independently with the spatial effect. Finally, a model which includes the four fixed effects that improve the ‘spatial only’ model was estimated (Table 5). We used the R-INLA Software, which uses a nested Laplace approximation, to estimate the different models. All models were fitted using uninformative priors for all the parameters and compared using the deviance information criterion (Table 6). A more precise description of a similar model can be found in ref. 21. Populations with at least one missing data point were removed before the analysis, resulting in a data set of 518 populations.

We then investigated the effect of the prevalence of co-infection on pathogen population growth rate (between July and September 2012) within local host populations. The prevalence of co-infection was estimated on a minimum sample size of four successfully genotyped individuals, and therefore nine populations were discarded from the analysis resulting in a final set of 135 populations. A two-step procedure was used to conduct this analysis. First, the change in the number of infected plants at the population level between the beginning and the end of the epidemic was modelled using a generalized linear mixed model framework using the function ‘lmer’ in the R package ‘lme4’ (refs 47, 48) with the percentage of co-infection, host plant coverage (m^2^), and pathogen-based connectivity^26^ of the population as fixed effects. Sub-networks were included in the model as random effects to account for any potential unmeasured spatially structured effects on within-population disease development. Sub-networks were defined using the hierarchic clustering algorithm described in ref. 26, accounting for both the size and relative spatial locations of the local pathogen populations, resulting in the classification of 135 populations into 45 different sub-networks. As a second step, because the host plant coverage (i) did not have a significant effect on the population growth rate, (ii) was partially redundant with pathogen-based connectivity and (iii) resulted in a decrease of the power of analysis due to missing data, we removed this fixed effect from the final model. For both the steps, *P* values were obtained by likelihood ratio tests of the full model against the model without the investigated effect.

## Author contributions

A.-L.L. conceived the project and obtained funding. A.-L.L., P.F.V. and H.S. conceived the experimental design, and H.S. carried out the experimental study. A-L.L., H.S. and B.B. designed large-scale genotyping scheme, B.B. oversaw genotyping and analysis of SNP data. H.S. and B.B. carried out statistical analyses. H.S. and A.-L.L. wrote the first draft of the manuscript and all the authors contributed to discussing the results and editing the manuscript.

## Additional information

**How to cite this article:** Susi, H. *et al.* Co-infection alters population dynamics of infectious disease. *Nat. Commun.* 6:5975 doi: 10.1038/ncomms6975 (2015).

## Supplementary Material

Supplementary Information Supplementary Figures 1-2 and Supplementary Tables 1-2

## Figures and Tables

**Table 1:** Factors explaining differences in disease load in the common garden experimental populations analysed with a generalized linear mixed model.

**Source**_ndf,ddf_	**F**	***P***
Pathogen treatment_2,617_	81.99	<0.0001
Days post inoculation_3,617_	213.65	<0.0001
Resistance type_2,46_	81.52	<0.0001
Genotype (resistance type)_14,46_	10.32	<0.0001
Pathogen treatment × days_6,617_	22.15	<0.0001
Pathogen treatment × resistance type_4,617_	10.67	<0.0001
Days post inoculation × resistance type_6,617_	29.93	<0.0001
Pathogen treatment × days post inoculation × resistance type_18,617_	3.84	<0.0001

ddf, denominator degrees of freedom; ndf, numerator degrees of freedom.

**Table 2:** Factors explaining differences in disease load of plant genotypes at 53 days post inoculation in the common garden experimental populations analysed with a generalized linear mixed model.

**Source**_ndf,ddf_	**F**	***P***
Co/Single treatment_1,54_	33.00	<0.0001
Resistance type_2,43_	31.66	<0.0001
Genotype (resistance type)_14,43_	4.43	<0.0001
Resistance type × co/single treatment_2,54_	6.64	0.0026

ddf, denominator degrees of freedom; ndf, numerator degrees of freedom.

**Table 3:** Differences in spore release in the trapping experiment analysed with a generalized linear mixed model.

**Source**_ndf,ddf_	**F**	***P***
Co/Single treatment_1,334_	23.16	<0.0001
Days post inoculation_4,334_	22.99	<0.0001
Genotype_7,24_	0.62	0.7374
Co/Single treatment × days after inoculation_4,334_	25.88	<0.0001

ddf, denominator degrees of freedom; ndf, numerator degrees of freedom.

**Table 4:** Differences in infection establishment on live leaves in the trapping experiment analysed with a generalized linear mixed model.

**Source**_ndf,ddf_	**F**	***P***
Co/Single treatment_1,338_	4.81	0.0290
Days post inoculation_1,338_	8.21	<0.0001
Genotype_7,24_	0.44	0.8678

ddf, denominator degrees of freedom; ndf, numerator degrees of freedom.

**Table 5:** Results of the Bayesian spatial logistic regression model analysing factors affecting the prevalence of co-infection in 641 *P. plantaginis* populations in Åland.

**Parameters name**	**Mean posterior estimate (posterior standard error)**
Intercept	−3.4 (±0.3)
Age of the population	0.36 (±0.11)
Pathogen-based connectivity	13.2 (±4.3)
Number of multilocus genotypes	0.15 (±0.04)
Disease abundance	0.33 (±0.07)
Host population area	NS
Road presence	NS
Distance to the shore	NS

NS, non-significant.

Non-significant parameters were removed from the models on the basis of deviance information criterion.

**Table 6:** Comparison of the different generalized linear mixed models tested for analysing factors affecting the prevalence of co-infection in 641 *P. plantaginis* populations in Åland.

**Model**	**DIC**	**Range posterior estimate (posterior standard error)**
Logistic regression	1,620	—
Spatial intercept	1,513	1,076 (±523)
Spatial logistic regression factor: age of the population	1,508	1,020 (±494)
Spatial logistic regression factor: pathogen-based connectivity	1,506	981 (±478)
Spatial logistic regression factor: number of multilocus genotypes	1,498	1,309 (±566)
Spatial logistic regression factor: disease abundance	1,492	1,253 (±570)
Spatial logistic regression factors: all	1,469	1,268 (±585)

DIC, deviance information criterion.

DIC used to determine the best model. Range represents the distance where the spatial autocorrelation drops below 0.1, according to the Matérn covariance function. The posterior estimates for the models including a spatial random effect are indicated.
