# Supplementary material for: Co-infection alters population dynamics of infectious disease
Source: Nat Commun. 2015 Jan 8;6:5975. doi: 10.1038/ncomms6975 (PMC4354079; doi:10.1038/ncomms6975)
Supplement: Supplementary Information — Supplementary Figures 1-2 and Supplementary Tables 1-2 [file ncomms6975-s1.pdf]

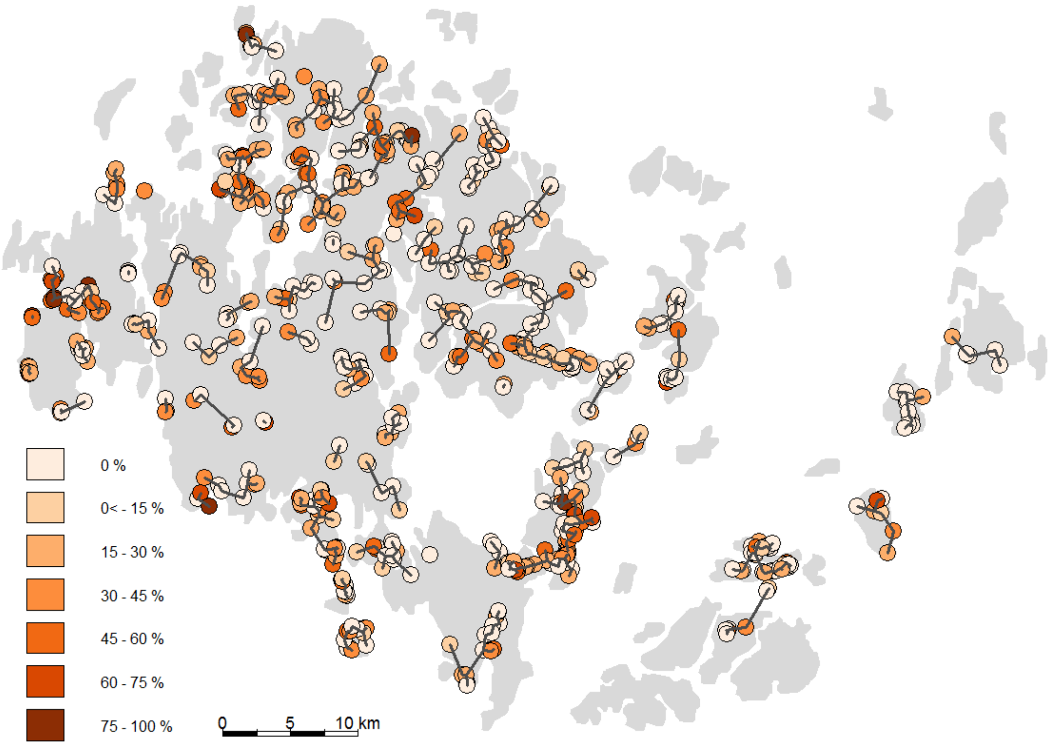

Supplementary Figure 1 | Map of the coinfection rate per population (patch). Each dot represents one of 641 populations sampled in 2012 in the Åland Islands. The color of the dot stands for the level of coinfection in the population. Populations that belong to the same sub-network are linked by a dark grey line.

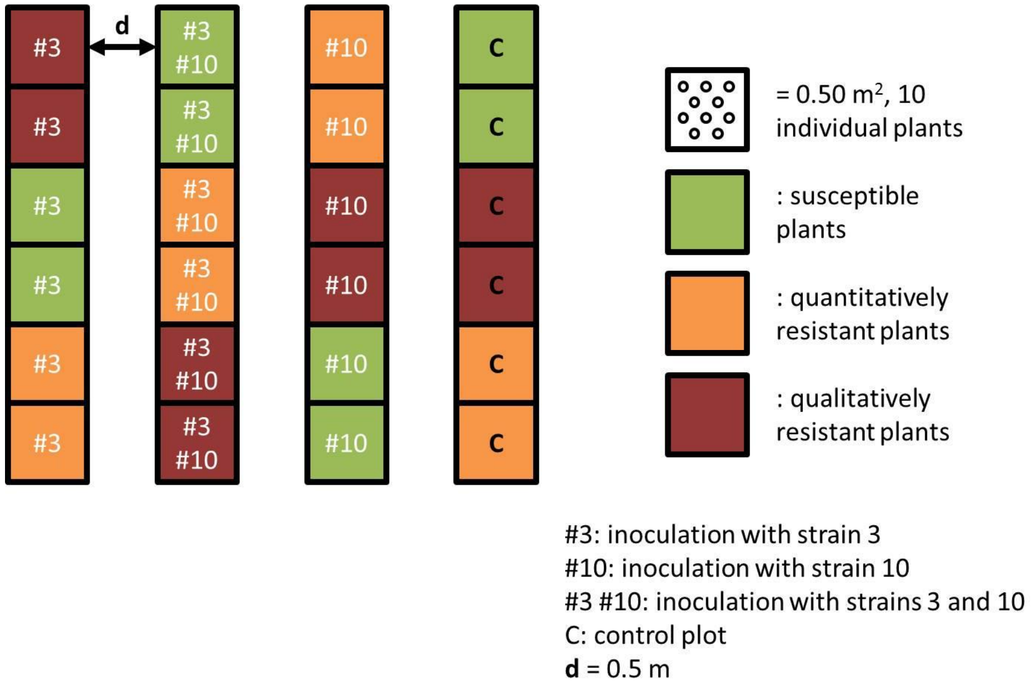

Supplementary Figure 2 | The common garden experimental design.

Supplementary Table 1| The *P. lanceolata* genotypes used in the experiments and their responses to *P. plantaginis* infection in the laboratory study.

				Qualitative resistance			Lesion development			Time to germination			Time to sporulation		
Plant ID	Resistance type	Clones used	Population ID	10	3	All strains	10	3	All strains	10	3	All strains	10	3	All strains
1	Qualitative	1	1413	0	0	0.71	4	3	3.50	4	4	4.00	7	11	9.00
2	Qualitative	5	1413	1	0	0.29	na	1	1.60	na	7	6.60	na	na	11.50
3	Qualitative	3	2220	0	1	0.57	2.5	na	2.83	6	na	7.00	12	na	10.67
4	Qualitative	4	2220	0	0	0.71	2.5	1	1.75	8	7	7.50	12	na	12.00
5	Qualitative	5	2220	0	0	0.29	2.5	1	2.00	4	7	6.00	8	na	10.33
6	Qualitative	2	511	0	1	0.71	1	na	1.00	10	na	7.50	na	na	na
7*	Quantitative	4	1413	0	0	0.14	2.5	1	1.50	5	4	4.83	12	na	12.00
8*	Quantitative	7	2220	0	0	0.00	2.5	2.5	2.00	4	6	6.43	12	11	11.50
9*	Quantitative	5	4	0	0	0.00	2.5	2.5	2.50	5	5	5.29	12	10	9.00
10*	Quantitative	4	4	0	0	0.00	1	2.5	2.36	6	9	6.71	na	11	10.40
11*	Susceptible	4	4	0	0	0.00	3	3	3.00	6	4	4.57	9	9	9.43
12	Susceptible	1	511	0	0	0.14	3	4	2.83	7	4	6.00	9	7	9.80
13*	Susceptible	2	511	0	0	0.00	3	4	3.43	7	4	5.14	10	7	7.14
14	Susceptible	1	511	0	0	0.00	3	3	3.00	5	5	6.43	8	9	9.57
15*	Susceptible	5	511	0	0	0.00	3	3	2.71	6	4	5.14	11	7	8.20
16	Susceptible	1	9031	0	0	0.00	3	4	3.57	4	4	4.57	8	8	8.00
17*	Susceptible	6	9031	0	0	0.00	4	4	2.86	4	4	5.14	7	7	9.17
18	Not used		1062	0	0	0.14	3	2.5	3.08	4	4	4.33	9	11	8.67
19	Not used		1062	0	0	0.00	3	3	3.07	5	6	5.14	9	10	9.00
20	Not used		1062	0	0	0.00	1	3	2.36	11	6	6.00	na	11	9.80
21	Not used		1062	0	1	0.14	1	na	1.67	6	na	6.83	na	na	10.00
22	Not used		1413	0	0	0.00	2.5	4	2.64	8	4	7.43	11	9	10.33
23	Not used		1413	0	0	0.00	2.5	3	3.14	5	6	5.29	12	10	9.29
24	Not used		1413	0	0	0.00	2.5	3	2.21	7	6	6.43	12	10	11.20
25	Not used		1413	0	0	0.00	3	3	3.07	4	6	4.71	9	9	9.29
26	Not used		1413	0	0	0.00	4	1	2.29	5	9	6.57	9	na	9.75
27	Not used		2220	0	0	0.00	1	4	2.00	11	4	6.57	na	7	8.67
28	Not used		2220	0	0	0.14	3	1	2.00	4	9	7.17	10	na	9.33
29	Not used		2220	0	1	0.14	1	na	1.67	6	na	5.83	na	na	9.00
30	Not used		2220	0	0	0.00	2.5	4	3.36	8	4	4.86	12	6	8.14
31	Not used		325	0	0	0.14	1	3	2.83	6	5	5.17	na	7	8.20
32	Not used		4	0	0	0.00	3	3	2.79	4	5	5.57	9	7	8.50
33	Not used		4	0	0	0.00	1	3	2.79	7	6	6.57	na	9	9.67
34	Not used		4	0	1	0.14	1	na	2.58	5	na	4.33	na	na	8.50
35	Not used		4	0	0	0.14	2.5	3	1.58	7	4	6.50	11	11	11.00
36	Not used		4	1	1	0.86	na	na	3.00	na	na	5.00	na	na	10.00
37	Not used		511	0	0	0.14	4	1	2.50	4	7	6.50	8	na	9.00
38	Not used		511	0	0	0.00	3	4	2.43	9	4	7.57	11	7	10.40
39	Not used		9031	0	0	0.00	3	2.5	2.43	4	6	5.86	8	8	8.60
40	Not used		9031	0	0	0.00	2.5	4	3.14	6	4	4.71	11	8	8.86
41	Not used		9031	0	0	0.14	2.5	4	3.00	6	4	6.17	11	8	10.00

The plant genotypes used in preliminary inoculation experiment (1-41 in the experimental populations (1-17) and in the spore trapping experiment (*). Resistance responses on laboratory experiment to strains used in the common garden experiments (strains 3 and 10 separately) and all seven strains used in the laboratory

experiment (average) are shown for each plant genotype. Infection success is used as a measure of qualitative resistance (0 = susceptible (infection) and 1 = resistance (no infection), and lesion development, time to germination and time to sporulation are used as measures of quantitative resistance.

Supplementary Table 2. Performance of *P. plantaginis* strains on *P. lanceolata* hosts with varying resistance strategies

	Strain ID	3	10	5	6	8	14	79
	Population ID	877	2821	689	689	228	9066	9609
	**Host type**							
**Infectivity**	Susceptible	1.00	1.00	1.00	1.00	0.86	1.00	1.00
	Quantitative	1.00	1.00	1.00	1.00	0.75	1.00	1.00
	Qualitative	0.67	0.83	0.33	0.50	0.17	0.33	0.33
	All	0.93	1.00	0.80	0.80	0.60	0.80	0.87
**Aggressiveness**	Susceptible	3.57	3.14	3.43	2.50	2.42	2.93	3.36
	Quantitative	2.13	2.13	1.00	2.38	2.00	2.13	3.00
	Qualitative	1.50	2.50	2.00	1.50	1.00	2.75	2.75
	All	2.75	2.80	2.58	2.33	2.28	2.67	3.15
**Time to germination**	Susceptible	4.14	5.57	5.00	5.86	5.83	5.71	4.86
	Quantitative	6.00	5.00	8.00	6.50	5.00	5.50	4.75
	Qualitative	6.25	6.40	6.00	7.33	6.00	6.50	6.00
	All	5.07	5.40	6.08	6.25	5.56	5.83	5.00
**Time to sporulation**	Susceptible	7.71	8.86	8.71	9.20	9.25	9.57	8.14
	Quantitative	10.67	12.00	na	10.67	10.00	8.50	10.00
	Qualitative	11.00	9.75	11.00	11.00	9.53	11.00	11.00
	All	8.82	9.79	9.00	9.75	9.50	9.40	9.08

The mean performance of a strain is shown on the qualitatively resistant, quantitatively resistant, and susceptible hosts that were used in the common garden experiment, and as the mean performance across all the 41 hosts used in the laboratory experiment. Infectivity of the strain is scaled as 0 = no infection and 1 = infection.
